# Predictive value of D-dimer to albumin ratio for severe illness and mortality in patients with COVID-19

**DOI:** 10.3389/fmed.2024.1410179

**Published:** 2024-07-31

**Authors:** Benjie Xiao, Zhangwei Yang, Huazheng Liang, Yudi Han, Yinyan Wu, Jingjing Xiao, Yong Bi

**Affiliations:** ^1^Department of Neurology, Zhoupu Hospital Affiliated to Shanghai University of Medicine & Health Sciences, Shanghai, China; ^2^Translational Research Institute of Brain and Brain-Like Intelligence, Shanghai Fourth People’s Hospital, School of Medicine, Tongji University, Shanghai, China; ^3^Medical Department, Shanghai Fourth People’s Hospital, School of Medicine, Tongji University, Shanghai, China; ^4^Suzhou Industrial Park Monash Research Institute of Science and Technology, Suzhou, Jiangsu, China; ^5^Department of Neurology, Shanghai Fourth People’s Hospital Affiliated to Tongji University School of Medicine, Shanghai, China

**Keywords:** COVID-19, D-dimer to albumin ratio, multivariate logistic regression, cox regression, prognosis

## Abstract

**Objective:**

Although the impact of the variants of COVID-19 on the general population is diminishing, there is still a certain mortality rate for severe and critically ill patients, especially for the elderly with comorbidities. The present study investigated whether the D-dimer to albumin ratio (DAR) can predict the severity of illness and mortality in COVID-19 patients.

**Methods:**

A total of 1,993 patients with COVID-19 were retrospectively reviewed and the association of DAR with severe or critical illness or death during hospitalization was analyzed. The area under the ROC curve was used to screen the best indicators, Chi-square test, rank sum test, and univariate and multivariate binary logistic regression analysis were used to calculate the mean value of difference and adjusted odds ratio (aORs) with their 95% CI, and finally, survival was analyzed using Kaplan–Meier (KM) curves.

**Results:**

Among 1,993 patients with COVID-19, 13.4% were severely ill, and the mortality rate was 2.3%. The area under the curve (AUC) using DAR to predict severe and critically ill patients was higher than that using other parameters. The best cut-off value of DAR was 21 in the ROC with a sensitivity of 83.1% and a specificity of 68.7%. After adjusting age, gender, comorbidities, and treatment, the binary logistic regression analysis showed that elevated DAR was an independent risk factor for severely ill and mortality of COVID-19 patients. The KM curve suggested that patients with a higher DAR was associated with worse survival. The negative predictive value of DAR (21) for adverse prognosis and death was 95.98 and 99.84%, respectively, with a sensitivity of 80.9 and 95.65%, respectively.

**Conclusion:**

The DAR may be an important predictor for severe illness and mortality in COVID-19 patients.

## 1 Introduction

While the COVID-19 is not treated as a pandemic any more, the virus is always present in the environment, and patients inflicted by this virus can be asymptomatic or with multiple organ failure or severe pneumonia. Although the impact of the variants of COVID-19 virus on the population is diminishing, there is still a certain mortality rate for severe or critically ill patients, especially for elderlies with comorbidities (1–3). Therefore, there is a need to investigate biomarkers that can be used to quickly predict the likelihood of acquiring a critical illness or death.

COVID-19 is a thrombo-inflammatory disease, which leads to hypercoagulation with alterations in hemostatic markers including elevated levels of the D-dimer due to endothelial injury. Consequently, the coagulation and fibrinolytic systems of the circulation system are activated (4–6). Multiple studies have shown that age and the increased level of the D-dimer are independent risk factors of death for severe or critically ill patients with COVID-19 (7–9).

Albumin is an essential transporter and drug-binding protein for various substances in the plasma and maintains the osmotic pressure of the blood (10). A decreased level of serum albumin among COVID-19 patients is associated with a higher incidence of adverse outcomes and a higher mortality rate (8, 11, 12). In this context, we retrospectively analyzed the clinical and laboratory data of the only designated hospital for the treatment of COVID-19 in Hongkou District of Shanghai at that time, compared the correlation between D-dimer, albumin and D-dimer to albumin ratio and the severe illness or death, and calculated the relevant OR values. Finally, we applied multiple models that may affect the prognosis for correction, the corrected OR values were calculated again to clarify whether DAR has a stronger predictive power for severe COVID-19 or mortality than other parameters.

## 2 Materials and methods

### 2.1 Study population and design

**Figure 1:**
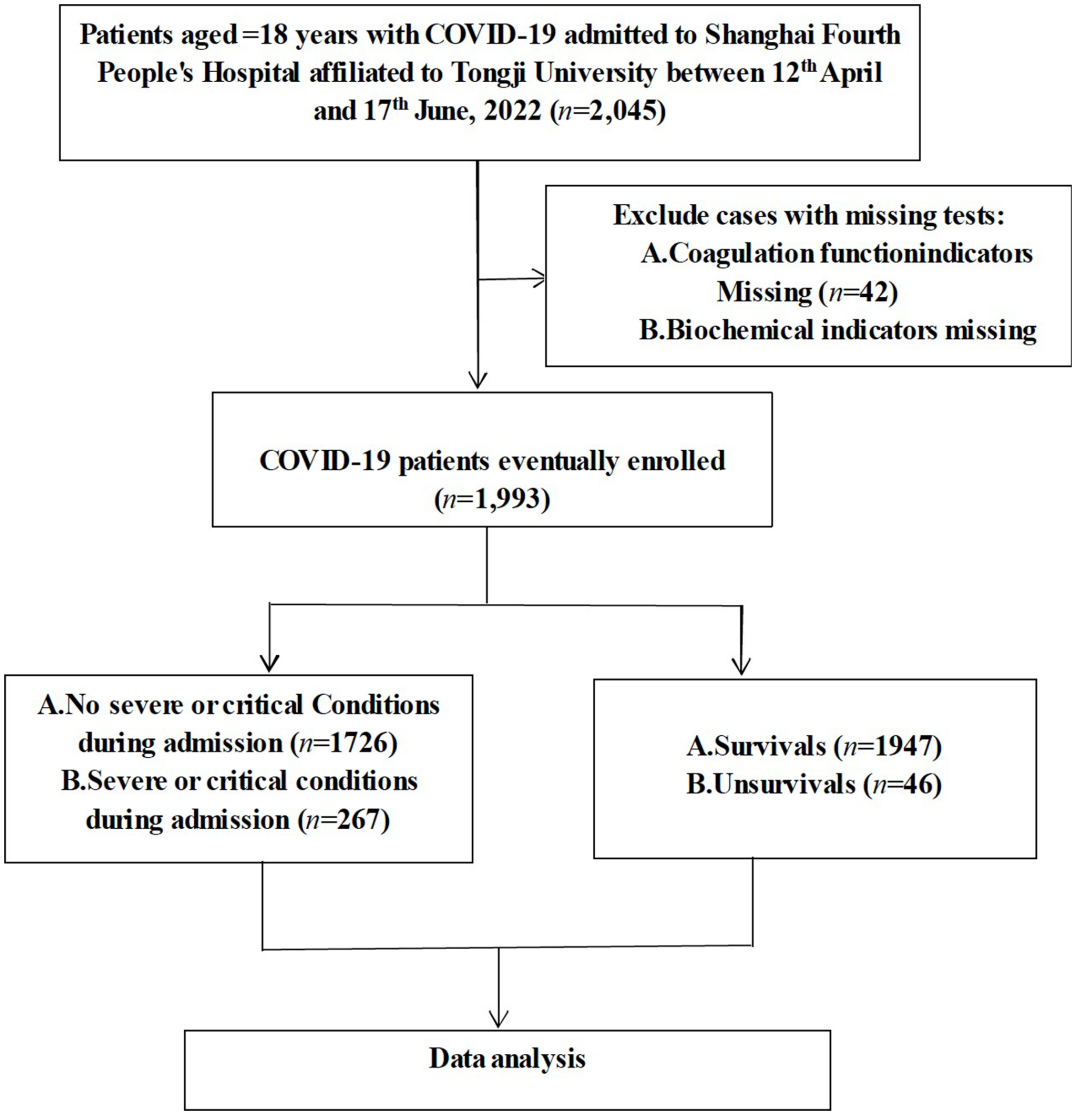
The flowchart of patient recruitment and data analysis.

This study was a single-center, retrospective, observational study on confirmed COVID-19 patients who were hospitalized to Shanghai Fourth People’s Hospital, School of Medicine, Tongji University from 12th April to 17th June, 2022. The diagnosis of COVID-19 was confirmed using the polymerase chain reaction (PCR) test. Patients with positive SARS-CoV-2 BA.2.2 PCR results were included in this study. Patients were excluded if they met any of the criteria below: (1) <18 years; (2) missing coagulation function indicators; and (3) missing biochemical indicators (Figure 1). This study was approved by the Human Ethics Committee of Shanghai Fourth People’s Hospital and written informed consent was waived as this study is a retrospective observational study.

### 2.2 Data collection

Both demographic and clinical data were collected from electronic medical records of hospitalized patients, including age, gender, comorbidities like hypertension, diabetes, coronary heart diseases, atrial fibrillation, history of stroke, results of blood tests, such as neutrophil counts, lymphocyte counts, platelet counts, the level of hemoglobin in the plasma, levels of serum C-reaction protein (CRP), procalcitonin, d-dimer, fibrinogen, alanine transaminase (ALT), aspartate aminotransferase (AST), creatinine, lactate dehydrogenase (LDH), total bilirubin, direct bilirubin, albumin, troponin-I. These were all obtained within 24 h of admission. Therapies included the antiviral therapy, anticoagulation, and the use of corticosteroid. All patients were closely monitored during their admission period and their outcomes, such as death or being discharged were recorded. The primary outcome of the present study was severe or critical illness or death during hospitalization.

The clinical genotyping criteria of COVID-19 virus were consistent with those of China’s official clinical guidelines. Critical or severe condition of COVID-19 was diagnosed if patients met any of the criteria below: (1) feeling short of breath, respiratory rate over or equal to 30 times/min; (2) saturation of blood oxygen equal to or less than 93% during breathing at rest; (3) the ratio of the arterial partial pressure of oxygen (PaO_2_) to the oxygen concentration (FiO_2_) < 300 mmHg; PaO_2_/FiO_2_ was corrected based on a formula for high altitudes (above 1,000 m): PaO_2_/FiO_2_ × [760/atmospheric pressure (mmHg)]; and (4) patient condition progressively deteriorated and lesions in the lungs significantly expanded by >50% within 24–48 h. Critical COVID-19 was diagnosed if patients presented with any of the following signs: (1) respiratory failure and the need for mechanical ventilation; (2) the appearance of shock; and (3) intensive care and treatment initiated due to other complications, such as organ failure other than respiratory failure.

### 2.3 Statistical analysis

Patients with missing core data were excluded from analysis as shown in Figure 1. Conditional Mean Completer was applied to other missing data. Continuous variables were expressed either in mean ± standard deviation (SD) or median or interquartile range (IQR), and categorical variables were displayed with absolute numbers or frequencies (%). Results were compared between groups using either independent sample t-tests, or Mann–Whitney U-tests for continuous variables, or Chi-square or Fischer’s exact tests for categorical variables. Severity of COVID-19 was assessed using the receiver operating characteristic (ROC) curve (13). The Youden index {Youden index = Sensitivity − (1 − specificity)} was adopted to validate screening results. The larger the Youden index is, the truer it is. Therefore, the largest Youden index was chosen as the cut-off value. Diverse models of the multivariate logistic regression were used to calculate the odds ratios (ORs), adjusted odds ratios (aORs), and their 95% confidence intervals (CIs) when correlating DAR with patient outcomes. Confounding factors adjusted in these models included those found in published literatures and those based on clinical assessment, especially variables that can render the relationship between DAR and clinical outcomes unclear, such as sex, age, comorbidities, treatment regimens, and blood assay markers. Meanwhile, survival was analyzed using Kaplan–Meier (KM) curves. Finally, the positive predictive value, negative predictive value, positive likelihood ratio, negative likelihood ratio and the diagnostic accuracy of DAR (with truncation value as the cut-off point) were calculated to evaluate the predictive value of DAR for the prognosis of COVID-19. Statistical analyses were conducted using the Statistical Package for the Social Sciences (SPSS version 24) and MedCalc version 22. *p* values <0.05 indicated statistical significance.

## 3 Results

### 3.1 Baseline characters of recruited patients

In the present study, a total of 2,045 patients were admitted to the hospital and 42 were excluded due to the lack of coagulation function biomarkers and another 10 due to lack of biochemical markers, resulting in the inclusion of 1,993 patients. The screening process was described in Figure 1, and the baseline characteristics of the included patients were presented in Table 1.

**Table 1:** Baseline characteristics of patients with adverse and non-adverse outcomes.

Variables	Total patients	Non-adverse	Severe or critical conditions	*p*-value
Patients, *n* (%)	1,993 (100%)	1,726 (86.6%)	267 (13.4%)	
Sex, *n* (%)				0.413
Female	1,158 (58.1%)	1,009 (58.5%)	149 (55.8%)	
Male	835 (41.9%)	717 (41.5%)	118 (44.2%)	
Age, median (IQR), years	76 (66, 87)	74 (65, 86)	86 (75, 90)	<0.001
Comorbidities, *n* (%)				
Hypertension	864 (43.4%)	720 (41.7%)	144 (53.9%)	<0.001
Diabetes	387 (19.4%)	332 (19.2%)	55 (20.6%)	0.600
Cardiovascular disease	36 (1.8%)	23 (1.3%)	13 (4.9%)	<0.001
Atrial fibrillation	80 (4.0%)	62 (3.6%)	18 (6.7%)	0.015
History of stroke	321 (16.1%)	224 (13.0%)	97 (36.3%)	<0.001
Laboratory testing				
Neutrophil count, ×10^9^/L	3.25 (2.29, 4.59)	3.11 (2.24, 4.33)	4.44 (2.88, 7.34)	<0.001
Lymphocyte count, ×10^9^/L	1.27 (0.89, 1.76)	1.34 (0.94, 1.81)	0.91 (0.59, 1.30)	<0.001
Platelet count, ×10^9^/L	178.00 (139.00, 225.00)	180.00 (142.25, 226.00)	166.00 (127.00, 212.00)	<0.001
Hemoglobin, g/L	126.00 (112.00, 136.00)	126.00 (114.00, 136.00)	120.00 (103.00, 133.00)	<0.001
C-reaction protein, mg/L	8.00 (2.81, 23.88)	6.62 (2.47, 17.26)	46.48 (13.34, 103.86)	<0.001
Procalcitonin, μg/L	0.02 (0.02, 0.06)	0.02 (0.02, 0.04)	0.11 (0.03, 0.41)	<0.001
Alanine transaminase, U/L	16.67 (11.88, 25.30)	16.83 (11.99, 25.28)	16.05 (9.80, 25.30)	0.099
Aspartate aminotransferase, U/L	23.62 (18.76, 31.21)	23.08 (18.50, 30.20)	27.42 (21.47, 44.04)	<0.001
Creatinine, μmoI/L	60.10 (48.90, 76.50)	59.80 (49.20, 74.78)	63.70 (45.80, 88.70)	0.212
Lactate dehydrogenase, U/L	188.44 (154.55, 224.42)	185.18 (152.55, 217.31)	226.04 (181.41, 289.50)	<0.001
Total bilirubin, μmoI/L	11.20 (8.26, 15.57)	11.28 (8.30, 15.57)	10.65 (7.92, 15.61)	0.229
Direct bilirubin, μmoI/L	2.71 (1.98, 3.98)	2.65 (1.94, 3.87)	3.15 (2.00, 4.97)	<0.001
Albumin, g/L	39.43 (36.25, 42.52)	39.88 (36.98, 42.97)	35.59 (32.10, 38.44)	<0.001
Troponin-I, μg/L	0.01 (0.01, 0.02)	0.01 (0.01, 0.02)	0.03 (0.02, 0.07)	<0.001
D-dimer, μg/L	560 (330, 1, 210)	500 (310, 960)	1, 510 (950, 2, 780)	<0.001
Fibrinogen, mg/dL	4.06 (3.65, 4.53)	4.05 (3.65, 4.50)	4.22 (3.67, 4.77)	<0.001
DAR, ×10^−6^	14 (8, 33)	13 (7, 26)	44 (25, 85)	<0.001
Therapies, *n* (%)				
Antiviral therapy	1,503 (75.4%)	1,291 (74.8%)	212 (79.4%)	0.104
Anticoagulation therapy	1,239 (62.2%)	1,021 (59.2%)	218 (81.6%)	<0.001
Use of corticosteroid	159 (8.0%)	57 (3.3%)	102 (38.2%)	<0.001

Take Adverse as the independent variable, the difference between univariate and multivariate logistic regression are Age, History of stroke, Lymphocyte count, C-reaction protein, Lactate dehydrogenase, Albumin, D-dimer, Anticoagulation therapy, Use of corticosteroids.

Among these patients, 267 (13.4%) demonstrated adverse outcomes. No significant difference in the percentage of male and female patients between the adverse-outcome and the non-adverse-outcome groups was found. However, the average age of patients in the adverse-outcome group was significantly higher than those in the non-adverse-outcome group (*p* < 0.01). The proportion of patients with hypertension, cardiovascular diseases, atrial fibrillation, and stroke was significantly higher in the adverse-outcome group compared to the non-adverse-outcome group (*p* < 0.05). Statistically significant differences were also found between both groups in the anticoagulant therapy and the use of corticosteroids but not in the antiviral therapy. Significantly lower levels of the lymphocyte count, platelet count, hemoglobin, alanine transaminase, and the total bilirubin were observed in the adverse-outcome group compared to the non-adverse-outcome group; whereas significantly higher levels of the neutrophil count, C-reactive protein, dimerized plasmin fragment D, potassium, aspartate transaminase, creatinine, and the lactate dehydrogenase were observed in the adverse-outcome group compared to the non-adverse-outcome group (*p* < 0.05).

### 3.2 Comparison of the ROC of the test biomarkers

**Figure 2:**
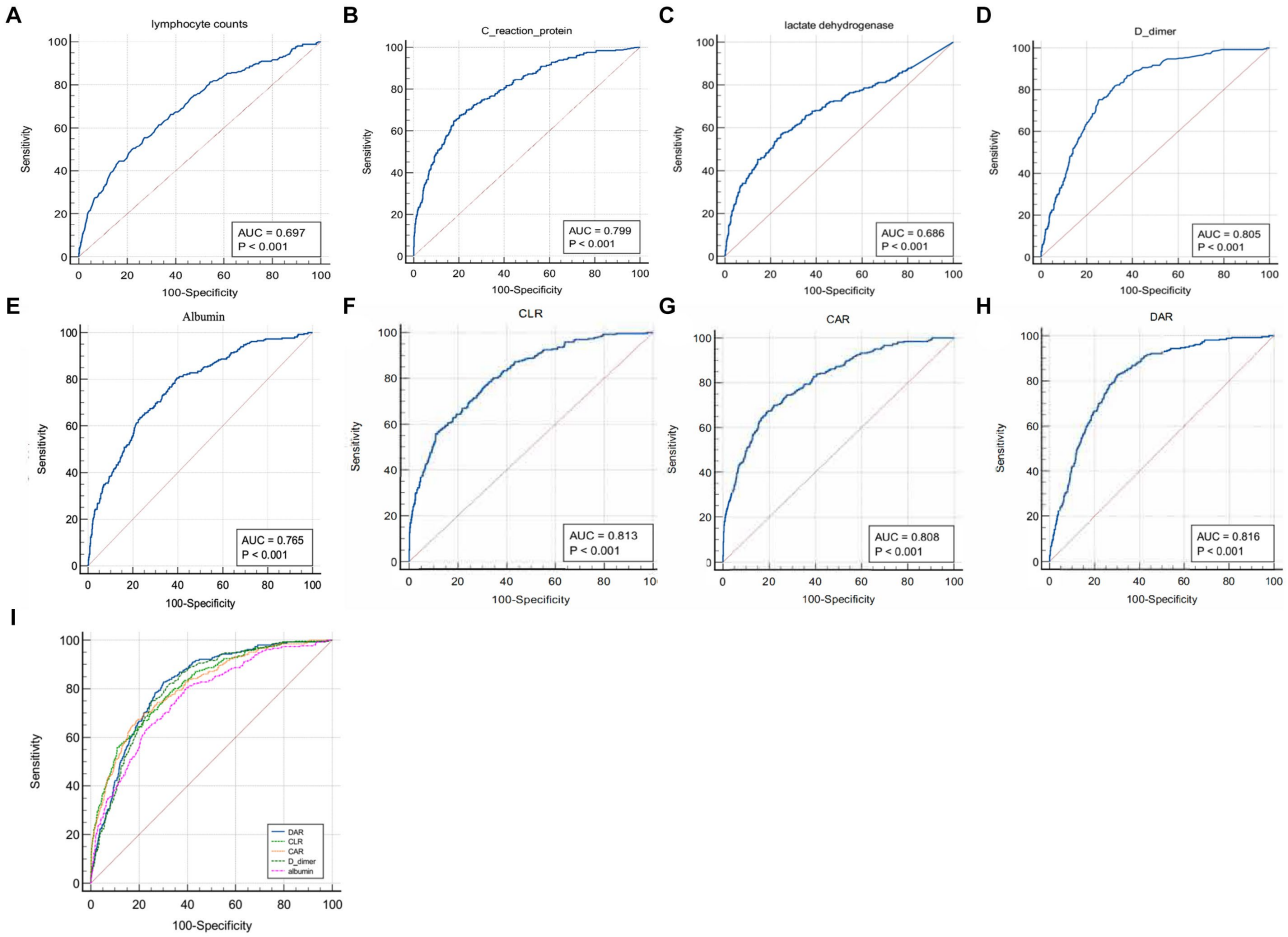
Comparison of areas under the ROC curve for indicators that were suggested as independent risk factors by univariate and multivariate regression analyses.

As shown in Tables 2, 3, the lymphocyte count, C-reactive protein, lactate dehydrogenase, D-dimer, albumin and DAR were likely to influence the capacity to predict adverse outcomes of COVID-19 patients. Based on the area under the ROC curve, it was found that the lymphocyte count, C-reactive protein, lactate dehydrogenase, D-dimer, albumin, C-reactive protein to lymphocyte ratio (CLR), C-reactive protein to albumin ratio (CAR) and the DAR had an ROC area of 0.697, 0.799, 0.686, 0.805, 0.765, 0.813, 0.808, and 0.816, respectively. Among them, DAR had the largest ROC area (Figure 2). When the cutoff value was 21%, the sensitivity was 83.1% and the specificity 68.7% in predicting adverse outcomes.

**Table 2:** Characteristics of ROC curves in COVID-19 patients (severe or critical conditions).

	ACU (95% CI)	SE	*p* value	Youden index	Cut-off value	Sensitivity	Specificity
Lymphocyte count	0.697 (0.677, 0.718)	0.018	<0.001	0.282	1.14	0.663	0.619
C-reaction protein	0.799 (0.780, 0.816)	0.015	<0.001	0.470	21.90	0.674	0.796
Lactate dehydrogenase	0.686 (0.665, 0.706)	0.02	<0.001	0.324	217.87	0.569	0.754
D-dimer	0.805 (0.787, 0.822)	0.013	<0.001	0.499	940.00	0.753	0.746
Albumin	0.765 (0.745, 0.783)	0.015	<0.001	0.408	38.83	0.802	0.607
CLR	0.813 (0.795, 0.830)	0.014	<0.001	0.458	10.26	0.783	0.675
CAR	0.808 (0.790, 0.825)	0.014	<0.001	0.480	0.62	0.670	0.809
DAR	0.816 (0.798, 0.833)	0.013	<0.001	0.518	21.00	0.831	0.687

CLR, C-reactive protein to lymphocyte ratio; CAR, C-reactive protein to Albumin ratio; DAR, D-dimer to Albumin ratio.

**Table 3:** Comparative analysis of area under indicator ROC curve (*Z*/*p* value).

	DAR vs. CLR	DAR vs. CAR	DAR vs. D-dimer	DAR vs. Albumin
*Z* value	0.193	0.471	5.592	3.304
*p* value	0.8470	0.6379	<0.0001	0.001

CLR, C-reactive protein to lymphocyte ratio; CAR, C-reactive protein to Albumin ratio; DAR, D-dimer to Albumin ratio.

### 3.3 Characteristics of patients with a higher or lower level than the cut-off value of DAR

In Table 4, recruited patients were divided into two groups based on the DAR cut-off (21%). A total of 724 patients showed their ROC greater than the cut-off value (high group) and 1,269 smaller than the cut-off value (low group), corresponding to 36.3 and 63.7% of the total number of patients, respectively. The proportion of males and females in the two groups was similar, but the average age of the high group was much higher than that of the low group (*p* < 0.01). In terms of comorbidities, significant differences were observed in hypertension, cardiovascular diseases, atrial fibrillation, and history of stroke (*p* < 0.05). Regarding treatments, statistically significant difference was found in the anticoagulant therapy and the use of corticosteroids, but not in the antiviral therapy (*p* < 0.05). In terms of laboratory tests, significant differences were observed in the neutrophil count, lymphocyte counts, platelet counts, C-reaction protein, procalcitonin, alanine transaminase, aspartate aminotransferase, lactate dehydrogenase, total bilirubin, direct bilirubin, troponin-I, albumin, d-dimer, and the DAR between these two groups (*p* < 0.05).

**Table 4:** Baseline characteristics of patients with DAR ≤ 21 and DAR > 21.

Variables	Total patients	DAR ≤ 21	DAR > 21	*p*-value
Patients, *n* (%)	1,993 (100%)	1,269 (63.7%)	724 (36.3%)	
Sex, *n* (%)				0.244
Female	1,158 (58.1%)	725 (57.1%)	433 (59.8%)	
Male	835 (41.9%)	544 (42.9%)	291 (40.2%)	
Age, median (IQR), years	76 (66, 87)	71 (62, 82)	85 (73, 90)	<0.001
Comorbidities, *n* (%)				
Hypertension	864 (43.4%)	505 (39.8%)	359 (49.6%)	<0.001
Diabetes	387 (19.4%)	236 (18.6%)	151 (20.9%)	0.220
Cardiovascular disease	36 (1.8%)	14 (1.1%)	22 (3.0%)	0.002
Atrial fibrillation	80 (4.0%)	39 (3.1%)	41 (5.7%)	0.005
History of stroke	321 (16.1%)	127 (10.0%)	194 (26.8%)	<0.001
Laboratory testing				
Neutrophil count, ×10^9^/L	3.25 (2.29, 4.59)	3.00 (2.12, 4.07)	3.76 (2.69, 5.68)	<0.001
Lymphocyte count, ×10^9^/L	1.27 (0.89, 1.76)	1.39 (1.00, 1.86)	1.11 (0.75, 1.49)	<0.001
Platelet count, ×10^9^/L	178.00 (139.00, 225.00)	178.00 (142.00, 221.00)	179.00 (134.25, 230.75)	<0.001
Hemoglobin, g/L	126.00 (112.00, 136.00)	129.00 (118.00, 139.00)	117.00 (103.00, 130.00)	0.993
C-reaction protein, mg/L	8.00 (2.81, 23.88)	5.39 (1.99, 13.07)	19.44 (6.31, 57.87)	<0.001
Procalcitonin, μg/L	0.02 (0.02, 0.06)	0.02 (0.02, 0.03)	0.04 (0.02, 0.15)	<0.001
Alanine transaminase, U/L	16.67 (11.88, 25.30)	17.31 (12.47, 25.59)	15.71 (10.31, 24.46)	<0.001
Aspartate aminotransferase, U/L	23.62 (18.76, 31.21)	22.94 (18.53, 29.29)	25.36 (19.21, 37.20)	<0.001
Creatinine, μmoI/L	60.10 (48.90, 76.50)	59.40 (49.30, 72.70)	61.30 (48.10, 88.50)	0.020
Lactate dehydrogenase, U/L	188.44 (154.55, 244.42)	181.57 (151.08, 209.78)	206.73 (167.13, 253.30)	<0.001
Total bilirubin, μmoI/L	11.20 (8.26, 15.57)	11.43 (8.41, 15.77)	10.98 (8.01, 15.25)	0.018
Direct bilirubin, μmoI/L	2.71 (1.98, 3.98)	2.66 (1.92, 3.86)	2.78 (2.00, 4.23)	0.006
Albumin, g/L	39.43 (36.25, 42.52)	40.77 (38.24, 43.71)	36.36 (33.35, 39.50)	<0.001
Troponin-I, μg/L	0.01 (0.01, 0.02)	0.01 (0.01, 0.02)	0.02 (0.01, 0.04)	<0.001
D-dimer, μg/L	560 (330, 1, 210)	0.38 (0.27, 0.54)	1.66 (1.13, 2.63)	<0.001
Fibrinogen, mg/dL	4.06 (3.65, 4.53)	4.04 (3.63, 4.45)	4.21 (3.67, 4.66)	<0.001
Therapies, *n* (%)				
Antiviral therapy	1,503 (75.4%)	968 (76.3%)	535 (73.9%)	0.234
Anticoagulation therapy	1,239 (62.2%)	750 (59.1%)	498 (68.8%)	<0.001
Use of corticosteroid	159 (8.0%)	46 (3.6%)	113 (15.6%)	<0.001

### 3.4 Association of DAR with adverse patient outcomes

As shown in Table 5, the percentages of adverse outcomes were 29.8 and 4.0%, respectively, in the high and low groups. The proportions of non-survival patients were 6.1 and 0.2%, respectively. These differences were statistically significant (*p* < 0.001).

**Table 5:** Association between DAR and clinical outcomes.

	DAR ≤ 21 (*n* = 1,269)	DAR > 21 (*n* = 724)	Chi-square value	*p* value
Adverse (*n* = 267)	51 (4.0%)	216 (29.8%)	264.796	<0.001
Non-adverse (*n* = 1,726)	1,218 (96%)	508 (70.2%)		
Survival (*n* = 1,947)	1,267 (99.8%)	680 (93.9%)	71.646	<0.001
Un-survival (*n* = 46)	2 (0.2%)	44 (6.1%)		

DAR, D-dimer to Albumin ratio.

### 3.5 Association of DAR with the risk of adverse outcomes

In Table 6, logistic regression models were used to evaluate the association between DAR (below/above the DAR cut-off) and the risk of developing adverse clinical outcomes as well as non-survivor clinical outcomes in COVID-19 patients.

**Table 6:** Correlation between DAR and the risks of adverse clinical outcomes.

	Adverse clinical outcomes			Non-survivor clinical outcomes		
	^a^OR	95% CI	*p* value	^a^OR	95% CI	*p* value
Unadjusted	10.155	7.358–14.015	<0.001	40.991	9.907–169.604	<0.001
Age, sex-adjusted (Model A)	7.734^a^	5.534–10.809	<0.001	32.254	7.615–136.614	<0.001
Multivariate1-adjusted (Model B)	7.027^a^	5.009–9.857	<0.001	30.899	7.256–131.581	<0.001
Multivariate2-adjusted (Model C)	5.839^a^	4.066–8.383	<0.001	21.243	4.864–92.780	<0.001
Multivariate3-adjusted (Model D)	3.412^a^	2.331–4.995	<0.001	16.773	3.808–73.881	<0.001

DAR, D-dimer to Albumin ratio.

Model A: adjusted for age and gender. Model B: adjusted for multivariable, including age, sex, hypertension, diabetes, coronary artery diseases, atrial fibrillation, history of stroke. Model C: including age, sex, hypertension, diabetes, coronary artery diseases, atrial fibrillation, history of stroke, antiviral therapy, anticoagulation therapy, use of corticosteroids. Model D: including age, sex, hypertension, diabetes, coronary artery diseases, atrial fibrillation, history of stroke, antiviral therapy, anticoagulation therapy, use of corticosteroids, C-reaction protein, Lymphocyte count, Lactate dehydrogenase. “a” indicates adjusted OR.

From the five models of regression analysis, it was found that DAR with a cut-off value of 21 was a potent predictor for adverse outcomes including non-survivor clinical outcomes. In terms of adverse outcomes, the unadjusted odds ratio (OR) was 10.155, and the adjusted OR (aOR) was 7.734 after adjusting age and gender (Model A), and the aOR of the adjusted models (Model B, Model C, and Model D) were 7.027, 5.839, and 3.412, respectively. The unadjusted odds ratio (OR) for non-survivor outcomes was 40.991, and the aOR after adjusting age and gender (Model A) was 32.254, and the aOR of the Model B, Model C, and Model D were 30.899, 21.243, and 16.773, respectively, suggesting that a large value of DAR was significantly associated with an increased risk of adverse outcomes.

### 3.6 The time-dependent risk of death among COVID-19 patients with a low or high DAR

**Figure 3:**
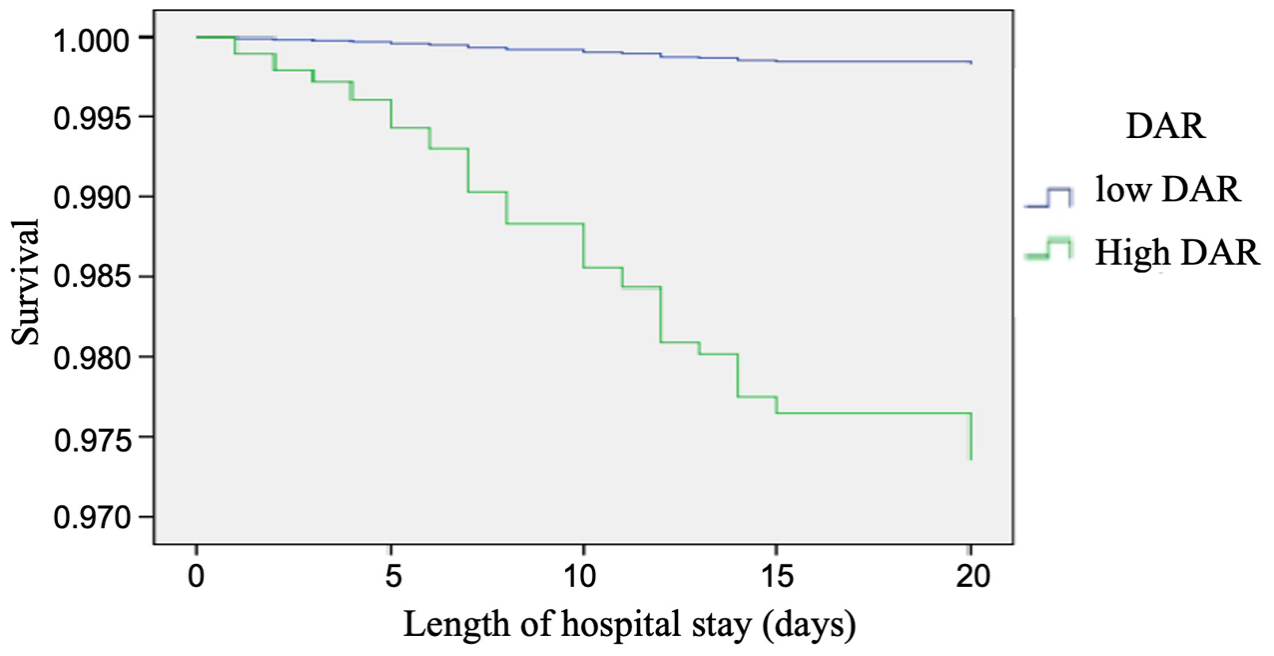
Cox regression analysis, the time-dependent risk of death in COVID-19 patients with low and high DAR using the Kaplan-Meier curve: Patients with a higher DAR showed a worse survival compared with those with a lower DAR (log rank *P* < 0.001).

In Figure 3, the time-dependent risk of death among COVID-19 patients with a low or high DAR was displayed using the Kaplan–Meier curve. It was found that patients with a higher DAR showed worse survival compared with those with a lower DAR (Log Rank *p* < 0.001).

In Tables 7, 8, the results indicated that the negative predictive values of DAR (21) for adverse prognosis and death were 95.98 and 99.84%, positive predictive values were 29.83 and 6.08%, respectively, with a sensitivity of 80.9 and 95.65%, and a specificity of 70.57 and 65.07%, respectively. The overall accuracy was 71.95 and 65.78%, respectively.

**Table 7:** Prediction value of DAR (21) for poor prognosis in patients with COVID-19.

Test item	Index value	95% CI
Sensitivity	80.90%	75.663–85.435%
Specificity	70.57%	68.355–72.710%
AUC	0.757	0.738–0.776
Positive likelihood ratio	2.749	2.503–3.018
Negative likelihood ratio	0.271	0.211–0.347
Positive predictive value	29.83%	27.915–31.827%
Negative predictive value	95.98%	94.904–96.838%
Accuracy	71.95%	69.923–73.916%

DAR, D-dimer albumin ratio; AUC, Area Under roc Curve.

**Table 8:** The predictive value of DAR (21) for death in patients with COVID-19.

Test item	Index value	95% CI
Sensitivity	95.65%	85.161–99.469%
Specificity	65.07%	62.910–67.194%
AUC	0.804	0.786–0.821
Positive likelihood ratio	2.739	2.512–2.986
Negative likelihood ratio	0.067	0.017–0.259
Positive predictive value	6.08%	5.602–6.590%
Negative predictive value	99.84%	99.391–99.959%
Accuracy	65.78%	63.650–67.864%

DAR, D-dimer albumin ratio; AUC, Area Under roc Curve.

## 4 Discussion

In the present study, severely ill patients accounted for 13.4%, and the mortality rate was 2.3% with a majority of patients being elderlies. The average age of adverse-outcome patients was over 10 years older than that of non-adverse-outcome patients. In addition, comorbidities like hypertension, cardiovascular diseases, atrial fibrillation, and history of stroke were more prevalent in adverse-outcomes patients than in non-adverse-outcome counterparts. Age and comorbidities remain important determinants of severe illness and mortality in COVID-19 patients. Laboratory tests showing the function of important organs and the status of the internal environment along with the choice of different treatment options (antiviral, anticoagulant, glucocorticoids, etc.) were also important factors determining the overall prognosis of patients. Previous literature reported that factors affecting the prognosis of COVID-19 included age, gender, comorbidities, abnormal laboratory indicators, etc. (14, 15), and rarely mentioned the influence of different treatment regimens on prognosis. Our study comprehensively analyzed the influence of confounding factors, such as age, gender, comorbidities, laboratory indicators, and different treatment regimens, on patient prognosis, which is one of the innovative features of our study.

To elucidate the predictive role of the target indicator DAR, we performed analysis on the area under the ROC curve for independent risk factors (including lymphocyte count, C-reactive protein, lactate dehydrogenase, D-dimer, albumin) and related ratios (including CLR, CAR, DAR), which were obtained through uni- or multi-factorial dual analysis. DAR was found to have the largest area under the curve. This is similar to a previous report on predicting COVID-19 mortality based on inflammatory markers, prognostic nutritional index (PNI) had the largest area under the curve to predict mortality, followed by CLR, and other indices (16). In addition, calibrated regression analysis and survival analysis were performed for each of the above factors that may impact their short-term outcomes. Results from these analyses were surprisingly consistent: DAR was an independent risk factor for developing severe illness or death in each model we analyzed.

Based on the area under the ROC curve, we found that DAR had the best effect on the short-term prognosis of COVID-19 patients at the cut-off value of 21 with a sensitivity of 0.831 and a specificity of 0.687. Furthermore, 29.8% of patients in the high DAR (>21) group were severely ill and 6.1% of them were dead, which were more than double their corresponding baseline rates. Pathophysiologically, COVID-19 patients are prone to hypercoagulability with increased D-dimer, which is related to activation of the coagulation system and insufficient blood volume due to systemic inflammatory response after infection. Multiple previous reports have reached the same conclusion (17–20). The decrease in albumin not only leads to the decrease of immunoglobulin production and the decrease of immunity, but also aggravates blood volume insufficiency, tissue edema and hypercoagulability (21). In addition, it has been reported that pulmonary capillary leakage syndrome plays a key role in the pathogenesis of severe COVID-19, and hypoalbuminemia may be a marker of the severity of pulmonary capillary endothelial injury in COVID-19 patients (22).

There is also evidence that advanced age, increased D-dimer and hypoalbuminemia are closely related to the high risk of thrombotic events in COVID-19 patients (23). The larger the inversely proportional ratio of the d-dimer against albumin, the more serious the condition of the patients is. The latter is closely related to the probability of death. Results of our study were consistent with previous findings.

Studies with a small sample size suggested that DAR at the early stage of COVID-19 might be an important parameter for predicting the likelihood of severe illness and mortality in the hospital (5, 24), which was supported by our study with a large number of patients.

Different reports have analyzed the predictive capacity of CLR, NLR, etc., on the disease and prognosis of COVID-19 patients, and reached different conclusions (25–28). The present study made a partial comparison in this respect. As mentioned above, DAR can not only reflect the pulmonary capillary function, but also the overall state of the body that changes in various aspects of the whole body, such as coagulation function, effective capacity and nutrient metabolism after inflammatory response. Therefore, it can better reflect the condition status of patients with novel coronavirus pneumonia than a single indicator or other composite indicators, such as CLR, NLR and CAR, etc., and has a more predictive effect on the prognosis of patients. So, it is worth paying attention in the future diagnosis and treatment process by clinicians with an aim to intervene early after detection and to reduce the incidence of severe illness and death.

In addition, we further evaluated the predictive value of DAR for the prognosis of COVID-19 by calculating the positive predictive value, negative predictive value and the accuracy. Results showed that the negative predictive value of DAR (21) for adverse prognosis and death was 95.98 and 99.84%, respectively, with a sensitivity of 80.9 and 95.65%, respectively. That is, the prediction value of DAR for adverse prognosis and mortality of COVID-19 patients is mainly negative prediction during screening and diagnosis.

### 4.1 Limitations

There are some limitations in our study. Firstly, our study is a single-center, retrospective observational study and there may be selection bias or other limitations that affect the extrapolation of our conclusions. Secondly, this study included more common comorbidities and treatment regimens, and did not include all regimens, nor did it include the duration of drug treatment. Therefore, it is likely that other comorbidities and therapeutics may affect the clinical outcome of COVID-19 patients. Thirdly, DAR was only evaluated for the first time at admission and did not assess the impact of dynamic changes in D-dimer and serum albumin on clinical outcomes. Despite these limitations, our results are consistent with previous studies, and our conclusions are based on a relatively large population and a large number of confounding factors. Future multi-center clinical studies with larger sample sizes and better design can further confirm our conclusion.

## 5 Conclusion

The D-dimer, serum albumin and DAR are useful parameters in predicting the occurrence of severe disease and death, and DAR has the highest efficiency. DAR at the cut-off value of 21 is an independent predictor of severe illness and death in COVID-19 patients, the prediction value of DAR for adverse prognosis and mortality of COVID-19 is mainly negative prediction during screening and diagnosis, and deserves adequate attention from clinicians in the course of diagnosis and treatment.

## Data availability statement

The original contributions presented in the study are included in the article/supplementary material, further inquiries can be directed to the corresponding author.

## Ethics statement

The studies involving humans were approved by the Human Ethics Committee of Shanghai Fourth People’s Hospital. The studies were conducted in accordance with the local legislation and institutional requirements. The participants provided their written informed consent to participate in this study.

## Author contributions

BX: Data curation, Formal analysis, Investigation, Methodology, Software, Writing – original draft. ZY: Formal analysis, Methodology, Software, Writing – original draft. HL: Project administration, Supervision, Validation, Writing – review & editing. YH: Data curation, Investigation, Software, Writing – original draft. YW: Data curation, Formal analysis, Software, Writing – original draft. JX: Data curation, Investigation, Software, Writing – original draft. YB: Conceptualization, Funding acquisition, Investigation, Supervision, Writing – review & editing.
